# Specification of epidermal cell fate in plant shoots

**DOI:** 10.3389/fpls.2014.00049

**Published:** 2014-02-25

**Authors:** Shinobu Takada, Hiroyuki Iida

**Affiliations:** Department of Biological Sciences, Graduate School of Science, Osaka UniversityToyonaka, Osaka, Japan

**Keywords:** epidermal cell differentiation, positional signal, HD-ZIP class IV transcription factor, cuticle, endosperm, receptor-like kinase, calpain-like cysteine protease, *Arabidopsis thaliana*

## Abstract

Land plants have evolved a single layer of epidermal cells, which are characterized by mostly anticlinal cell division patterns, formation of a waterproof coat called cuticle, and unique cell types such as stomatal guard cells and trichomes. The shoot epidermis plays important roles not only to protect plants from dehydration and pathogens but also to ensure their proper organogenesis and growth control. Extensive molecular genetic studies in *Arabidopsis* and maize have identified a number of genes that are required for epidermal cell differentiation. However, the mechanism that specifies shoot epidermal cell fate during plant organogenesis remains largely unknown. Particularly, little is known regarding positional information that should restrict epidermal cell fate to the outermost cell layer of the developing organs. Recent studies suggested that certain members of the HD-ZIP class IV homeobox genes are possible master regulators of shoot epidermal cell fate. Here, we summarize the roles of the regulatory genes that are involved in epidermal cell fate specification and discuss the possible mechanisms that limit the expression and/or activity of the master transcriptional regulators to the outermost cell layer in plant shoots.

## Introduction

The shoot epidermis is a single layer of surface cells that are morphologically characterized by anticlinal cell division patterns. The outer surface of the shoot epidermis is covered with a hydrophobic structure called a cuticle, which prevents water loss, pathogen attacks, and post-genital fusion of organs (Yeats and Rose, 2013). Besides basic pavement cells, leaf epidermis contains specialized cell types such as hair cells (trichomes) and stomatal guard cells, which function to cope with dehydration and pathogen attacks. In addition to its protective function, the epidermis plays roles in the regulation of organ growth and shoot stem cell maintenance (Savaldi-Goldstein et al., 2007; Knauer et al., 2013; Nobusawa et al., 2013). In addition, protodermal cells in the shoot meristem and the embryo are necessary for the production and transport of the phytohormone auxin, which drives embryonic axis formation and lateral organ primordia initiation (Reinhardt et al., 2003; Kierzkowski et al., 2013; Robert et al., 2013; Wabnik et al., 2013).

Determination of shoot epidermal fate relies on a “position” rather than a cell “lineage,” as clonal analyses has shown that there is no strict lineage restriction in developing leaves; cells can flexibly change their fates, and only the cells finally located at the surface of the organ develop into epidermis (Stewart and Dermen, 1975). However, positional cues that determine shoot epidermal cell fate remain largely unknown. This review describes recent advances in the studies of epidermal cell specification in the shoots, focusing mainly on the regulation of key transcription factors.

## HD-ZIP class IV transcription factors are key regulators for epidermal cell specification

**Figure 1:**
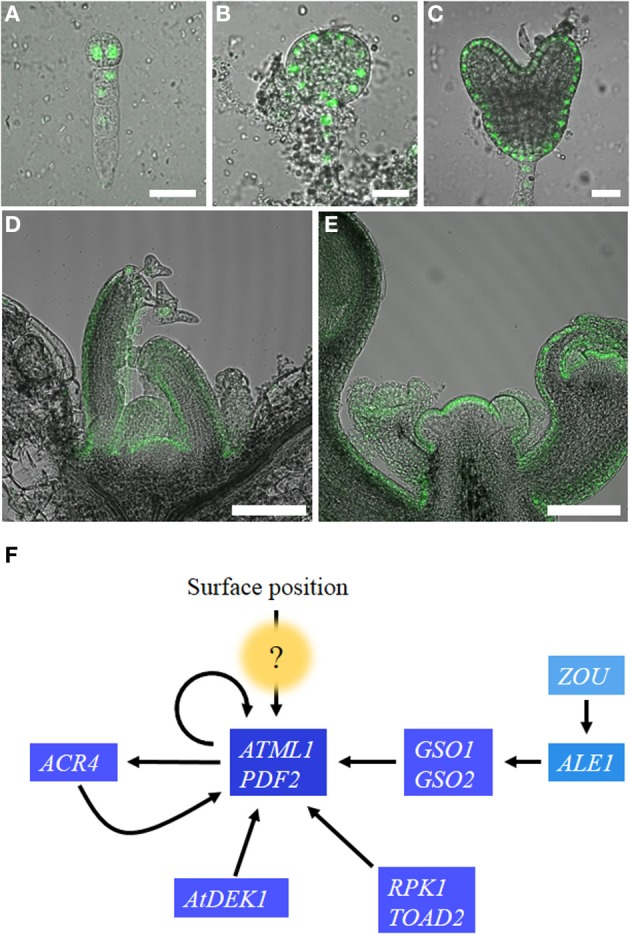
**Regulation of *ATML1* expression in the embryos and shoot apices**. *ATML1* promoter activity visualized using a nuclear-localized GFP reporter in the developing embryos **(A–C)** and the shoot apices **(D,E)**. Two-cell stage **(A)**, early globular stage **(B)**, and heart stage **(C)** embryos are shown. *GFP* expression is restricted to the outermost cell layer after the 16-cell stage. Epidermis-specific expression is observed in vegetative **(D)** and inflorescence **(E)** shoot apices. **(F)** Genetic interactions among genes involved in epidermal cell fate specification during *Arabidopsis thaliana* embryogenesis. Arrows indicate positive interactions. The question mark indicates currently unknown components. For details, please see the main text. Scale bars, 20 μm in **(A–C)**; 100 μm in **(D,E)**.

*ARABIDOPSIS THALIANA MERISTEM L1 LAYER* (*ATML1*) was identified as an epidermis-specific homeobox gene that belongs to the HD-ZIP class IV family (Lu et al., 1996). *ATML1* expression is first detected in the embryos as early as the one-cell stage and its expression is restricted to the outermost cells around the 16-cell stage (dermatogen stage) after the embryos have undergone tangential cell divisions to generate outer protodermal cells and inner cells (Lu et al., 1996; Sessions et al., 1999; Takada and Jürgens, 2007; Figures 1A–E).

Mutations in *ATML1* and its closest homolog *PROTODERMAL FACTOR2* (*PDF2*) caused severe phenotypes associated with defects in epidermal cell specification (Abe et al., 2003). Strong mutant alleles of *atml1;pdf2* showed embryo-lethal phenotypes with irregular division patterns of the protoderm, whereas weak mutant alleles of *atml1;pdf2* produced a few leaves that lack an epidermis (Abe et al., 2003; San-Bento et al., 2014; Supplementary Table 1). *ATML1* homologs have been isolated in several species and most of them are preferentially expressed in the epidermis (Ito et al., 2003; Nakamura et al., 2006; Javelle et al., 2011). Several of these genes are implicated in epidermis-related functions, not only for the initial specification of surface cell fate but also for the generation of distinct cell types within the epidermis (Rerie et al., 1994; Roeder et al., 2012; Peterson et al., 2013; Takada, 2013).

HD-ZIP class IV transcription factors may function as transcriptional activators or repressors (Ohashi et al., 2003; Yu et al., 2008; Javelle et al., 2010; Depège-Fargeix et al., 2011; Peterson et al., 2013). ATML1 and PDF2 were shown to bind *in vitro* to an 8-bp sequence called the L1 box (Abe et al., 2001, 2003). Considering that an L1 box is often found in the promoters of epidermis-specific genes including *ATML1* and *PDF2*, ATML1 and PDF2 have been proposed to positively regulate the expression of epidermis-specific genes (Abe et al., 2001, 2003). In fact, expression of epidermis-related genes was decreased in *atml1;pdf2* (Abe et al., 2003; Takada et al., 2013). Moreover, gain-of-function experiments suggest that *ATML1* activates expression of several epidermis specific genes during the initiation of new epidermal cell fate, but may also function as a negative regulator in maintaining expression levels (Takada et al., 2013; San-Bento et al., 2014). Notably, overexpression of *ATML1* was sufficient to induce differentiation of epidermal cells such as stomata and trichomes in the inner tissues of leaves (Takada et al., 2013; Supplementary Table 1). These results are consistent with the idea that ATML1 is a master transcriptional regulator for epidermal cell specification in shoots.

Importantly, expression of *ATML1* and its putative orthologs depends on a “surface” position, irrespectively of epidermal cell identity or cell lineage, as indicated by the presence of *ATML1* promoter activity at the surface mesophyll cells of *atml1;pdf2* leaves (Takada et al., 2013). In addition, expression of *RICE OUTERMOST CELL-SPECIFIC GENE1* (*ROC1*), a rice HD-ZIP class IV gene, was induced on the cut surface during callus regeneration (Ito et al., 2002). In the maize *extra cell layers1* (*xcl1*) mutant, which develops multilayered epidermis by amplification of differentiated epidermal cells, expression of an HD-ZIP class IV gene was detected only in the outermost epidermal layer (Kessler et al., 2002). These reports suggest that identification of upstream regulators that determine the outermost cell-specific expression of *ATML1* homologs would be an effective strategy for identifying positional signals that specify shoot epidermal cell fate.

## Positional signals that specify epidermal cell fate

Deletion and mutational analyses of an *ATML1* promoter revealed the involvement of several positive regulators in the protoderm-specific activation of *ATML1* (Takada and Jürgens, 2007). MicroRNAs and phytohormone auxin, two major components that are known for morphogen-like activity, appeared not to be involved in the outermost cell-specific expression of *ATML1* (Takada and Jürgens, 2007; Nodine and Bartel, 2010). Below, we discuss the candidate molecules or genes that may provide positional cues for shoot epidermal cell specification (Figure 1F).

### Cuticle

Cuticle is a hydrophobic lipid layer formed on the surface of the shoot epidermis. In cuticle-deficient plants, trichome numbers, stomatal density, and regular anticlinal cell division of the epidermis are impaired, suggesting that cuticular components and/or their precursors are required for the patterning of epidermis (Yephremov et al., 1999; Gray et al., 2000; Sieber et al., 2000). Cuticle can be observed in the zygote and is maintained only in the outermost cells of the embryo even before a layer of protoderm is visible (Bruck and Walker, 1985). Therefore, the presence of cuticle or cuticle biogenesis may be instructive for epidermal cell specification and/or maintenance. In fact, expression of *ROC1* was reduced in a rice mutant defective in the biosynthesis of very-long-chain fatty acids (VLCFAs), which serve as precursor of cuticular wax (Tsuda et al., 2013). Although it has been shown that *ATML1* and other HD-ZIP class IV genes positively regulate expression of cuticle biosynthesis genes and facilitate cuticle deposition, these results suggest that cuticle also functions to maintain epidermal cell identity as a positive feedback mechanism (Javelle et al., 2010; Wu et al., 2011; Takada, 2013; Takada et al., 2013). VLCFAs or its derivatives produced in the epidermis have been recently suggested to function as non-cell autonomous signals that promote cell proliferation in internal tissues (Nobusawa et al., 2013). Therefore, some intermediates or byproducts formed during cuticle biosynthesis may play roles in pattern formation in plants.

### Signaling from the endosperm

In the angiosperms, the embryo is surrounded by endosperm tissues, which provide nutrients to the developing embryo. Recent studies show that signaling from the endosperm is necessary for epidermal cell differentiation during embryogenesis. *abnormal leaf shape1* (*ale1*) and *zhoupi* (*zou*) mutants are defective in cuticle formation in organs generated during embryogenesis (Tanaka et al., 2001; Yang et al., 2008; Supplementary Table 1). *ZOU* encodes an endosperm-specific transcriptional regulator that promotes the expression of *ALE1* and other genes required for the breakdown of the endosperm (Yang et al., 2008; Supplementary Table 1; Figure 1F). *ALE1* encodes a subtilisin-like serine protease, and its expression in the endosperm is sufficient to rescue cuticle-deficient phenotypes of *ale1* and *zou*, suggesting that *ALE1* non-cell-autonomously promotes epidermal cell differentiation (Tanaka et al., 2001; Xing et al., 2013; Supplementary Table 1; Figure 1F). Considering that subtilisin-like proteases are involved in the processing of peptide hormone precursors in animals, the simplest scenario would be that ALE1 produces ligands promoting epidermal cell specification in the endosperm and the outermost cells of the embryos that receive those ligands differentiate into the epidermis (Steiner, 1998; Tanaka et al., 2001). GASSHO1 (GSO1) and GSO2 are candidate receptor-like kinases that receive signals produced by ALE1. GSO1 and GSO2 are preferentially expressed in the embryo, and *gso1;gso2* shows severe cuticle-deficient phenotypes epistatic to those of *ale1* (Tsuwamoto et al., 2008; Xing et al., 2013; Supplementary Table 1; Figure 1F).

It is not certain whether ALE1 and GSO1/GSO2 are generally required for the initiation of epidermal cell fate or specifically required for the cuticle formation on the surface of the embryo. In fact, epidermal cell specification can occur in the absence of the endosperm (such as during organ regeneration from calli, somatic embryogenesis, and aerial organ initiation in post-embryonic development). Plant embryos may require ALE1 and GSO1/GSO2-mediated signaling for efficient deposition of cuticle on the surface of the protodermal cells that develop in close physical contact with surrounding endosperm cells.

### CRINKLY4

*crinkly4* (*cr4*) is a maize mutant with defects in the development of leaf epidermis and aleurone layer (Becraft et al., 1996). *CR4* encodes a plant-specific receptor-like kinase, and mutations in a homolog of *CR4* in *Arabidopsis* [*ARABIDOPSIS THALIANA HOMOLOGUE OF CRINKLY4* (*ACR4*)] cause phenotypes defective in epidermal cell differentiation, lateral root initiation, and root initial cell maintenance (Gifford et al., 2003; Watanabe et al., 2004; De Smet et al., 2008; Stahl et al., 2009, 2013; Supplementary Table 1). Although *acr4* shows a mild effect on epidermal cell differentiation, *acr4;ale1* double mutants show severe phenotypes with reduced *ATML1* expression in the embryo, suggesting that *ACR4* acts in parallel with the “endosperm pathway” to positively regulate epidermal cell differentiation upstream of *ATML1* (Tanaka et al., 2007; Supplementary Table 1; Figure 1F).

However, expression of *ACR4* is restricted to the epidermis of the embryos and shoots, and its epidermis-specific expression depends on an L1 box in the promoter, suggesting that *ACR4* is a downstream target of HD-ZIP class IV transcription factors (Tanaka et al., 2002; Gifford et al., 2003; San-Bento et al., 2014; Supplementary Table 1; Figure 1F). *ACR4* expression consistently began later than *ATML1* expression during embryogenesis (Tanaka et al., 2002; Gifford et al., 2003). Moreover, ATML1 and PDF2 were associated with an *ACR4* promoter *in planta*, and *ACR4* expression was reduced in *atml1;pdf2* (Abe et al., 2003; San-Bento et al., 2014).

ACR4 is localized to the basal and lateral membranes of the epidermis, suggesting that it is involved in intercellular communication between the same and different layers (Gifford et al., 2003, 2005; Watanabe et al., 2004). ACR4 and CR4 were shown to localize to plasmodesmata (PD), pores connecting plant cells, in the aleurone cells and the cotyledon epidermis (Tian et al., 2007; Stahl et al., 2013). Pore sizes of the PD connecting aleurone cells were wider than those connecting aleurone and underlying starchy endosperm cells (Tian et al., 2007). These observations may suggest that CR4 is involved in the modulation of the size of PD pores and facilitates intercellular communication between the same layer, maintaining epidermal/aleurone cell fate. Considering together, these results suggest that ACR4 is possibly more involved in the maintenance of epidermal cell fate downstream of *ATML1* than in the perception of positional signals for epidermal cell specification (Figure 1F).

### Leucine-rich repeat receptor-like kinase in the embryo

RECEPTOR-LIKE PROTEIN KINASE1 (RPK1) and TOADSTOOL2 (TOAD2) are leucine-rich repeat receptor-like kinases redundantly required for epidermal cell differentiation in the embryo (Nodine et al., 2007). *rpk1;toad2* shows embryo-lethal phenotypes with disorganized cell division patterns particularly in the basal half of the embryo proper (Nodine et al., 2007; Supplementary Table 1). *ATML1* mRNA was detected in the outer cell layer of *rpk1;toad2* at the dermatogen stage but disappeared after the early globular stage, suggesting that these receptor-like kinases are necessary for the maintenance but not for the initial specification of epidermal cell fate (Nodine et al., 2007; Supplementary Table 1; Figure 1F). Because of the embryonic lethality, the roles of RPK1 and TOAD2 in shoot epidermal cell differentiation in the post-embryonic development are unknown.

### Defective kernel 1: a calpain-like protease

DEFECTIVE KERNEL 1 (DEK1) is a calpain like cysteine protease that is conserved among land plants (Lid et al., 2002; Liang et al., 2013). Downregulation of *DEK1* expression in several species causes phenotypes associated with defects in epidermal differentiation such as reduced deposition of cuticle, disorganization of cell division planes, and ectopic differentiation of mesophyll plastids (chloroplasts) in the surface cells (Becraft et al., 2002; Ahn et al., 2004; Johnson et al., 2005; Tian et al., 2007; Supplementary Table 1). Strong mutant alleles of *dek1* cause embryo-lethal phenotypes in *Arabidopsis*, maize, and rice, and expression of *ATML1* homologs disappears in these embryos, implying that *DEK1* is necessary for the initiation of epidermal fate in the early embryo (Lid et al., 2002, 2005; Johnson et al., 2005; Hibara et al., 2009; Supplementary Table 1; Figure 1F).

*DEK1* mRNA is expressed ubiquitously, suggesting that its activity is regulated post-translationally (Wang et al., 2003; Johnson et al., 2005; Lid et al., 2005; Hibara et al., 2009). DEK1 is composed of an N-terminal membrane-spanning region and a C-terminal cytosolic region that includes the calpain cysteine protease (Lid et al., 2002; Tian et al., 2007). It has been hypothesized that upon binding of epidermis-promoting ligands to the N-terminal region, DEK1 is cleaved by its autocatalytic activity to release the C-terminal region, an active form of the calpain (Wang et al., 2003; Tian et al., 2007; Johnson et al., 2008). In animals, calpain-like proteases are involved in the activation/inactivation of several signaling molecules, suggesting that DEK1 transduces signals for epidermal specification (Storr et al., 2011). However, overexpression of active truncated forms of DEK1 in *Arabidopsis* was not sufficient to upregulate the expression of *ATML1* (Johnson et al., 2008; Supplementary Table 1). Moreover, modulation of *DEK1* activity affected cell division and growth also in internal tissues, suggesting that the action of DEK1 is not epidermis-specific (Johnson et al., 2008; Supplementary Table 1). Johnson et al. (2008) proposed that DEK1 controls mainly cell division in developing leaves and that epidermal cells respond more sensitively to the amount of DEK1; reduction in cell division rate in *dek1* may cause discontinuity and abortion of the epidermis (Johnson et al., 2008). In this scenario, DEK1 is possibly more involved in the maintenance rather than the initiation of the epidermal cell layer.

### Post-transcriptional regulation of HD-ZIP class IV activity

Localization of some HD-ZIP class IV transcription factors was not limited to nuclei of heterologous cells (Zhang et al., 2010; Yang et al., 2011). The HD-ZIP class IV transcription factor GLABRA2 (GL2), a positive regulator of trichome formation, was restricted to the nuclei only in trichome cells and not in internal cells, suggesting a cell-type-specific regulation of nuclear transport (Szymanski et al., 1998). HD-ZIP class IV transcription factors contain a putative lipid/sterol binding domain (START) and a dimerization motif (ZLZ), implying a regulation of their activities by dimerization and binding of lipid/sterol ligands (Schrick et al., 2004). In fact, sterol and VLCFA biosynthesis-deficient mutants are defective in proper distribution of stomata and trichomes, respectively, (Yephremov et al., 1999; Qian et al., 2013). However, to date the roles of START domains have not been investigated, except for an observation showing that an N-terminal part of a START domain can function as a transcriptional activation domain in yeast and maize suspension cells (Depège-Fargeix et al., 2011).

ATML1 was shown to heterodimerize with PDF2 *in planta* and it is possible that dimerization with other HD-ZIP class IV proteins changes the activity of ATML1 in a cell-type dependent manner, although ectopic expression of *ATML1* alone was sufficient to induce epidermal cell fate in inner tissues (Takada et al., 2013; San-Bento et al., 2014). In related transcription factors, DNA-binding was inhibited by oxidation of Cys residues in the ZLZ motif, suggesting that redox signals are also involved in the regulation of ATML1 activity (Tron et al., 2002).

## Repression of inner cell fate

Several lines of evidence show that acquisition of epidermal cell fate is associated with a loss of mesophyll or internal cell fate. First, epidermis-deficient *atml1;pdf2* and *DEK1* knockdown lines showed ectopic differentiation of mesophyll cells on the surfaces of leaves and cotyledons, respectively, (Abe et al., 2003; Johnson et al., 2005). Second, *rpk1;toad2* embryos exhibited ectopic subepidermal marker expression in the outermost cell layer (Nodine et al., 2007; Supplementary Table 1). Third, overexpression of *ATML1* decreased differentiation of green mesophyll cells in leaves (Takada et al., 2013). Moreover, *cr4* and *dek1*, which are defective in “surface” aleurone layer differentiation in the maize endosperm, cause ectopic differentiation of “inner” starchy endosperm cells on the surface of the endosperm (Becraft et al., 1996; Becraft and Asuncion-Crabb, 2000). Therefore, repression of internal or “default” cell fate may be a general requirement for surface cell differentiation in plants. It is not possible, however, to test whether or not mesophyll cell differentiation represses epidermal cell fate because no positive regulators of inner cell fate are available at present. Chloroplast development itself appears not to exert a negative effect on epidermal cell differentiation, considering that stomatal guard cells possess chloroplasts and no ectopic epidermal cell differentiation has been reported in the mesophyll tissues of albino plants (Stewart and Dermen, 1975).

Mesophyll cells possibly represent a primitive state of leaf cells, considering that ancestral aquatic algae are composed mainly of mesophyll-like cells. Land plants may have repressed mesophyll cell differentiation to evolve an epidermis on the surface. Evolutionary studies, including comparative genomics, may be useful for identifying molecular components that promote epidermal cell formation (Zalewski et al., 2013).

## Future perspectives

Despite the extensive molecular genetic studies in model plants, positional signals that specify shoot epidermal cell fate remain unknown (Figure 1F). Most of the receptor-like kinases, characterized by their roles in shoot epidermal cell differentiation, are possibly involved in the maintenance than in the specification of epidermal cell fate. This appearance may be because of the difficulties in distinguishing between phenotypes associated with “specification” and those related to “maintenance” of epidermal cell fate in forward genetic screens.

The cuticle-bearing outermost cells should have distinct mechanical properties compared with inner cells. Moreover, cells located at the surface are unique, in that they are in constant contact with the environment. These unique properties could influence the differentiation of epidermal cells. Attempts to directly isolate epidermis-promoting biomolecules and to identify physical/environmental constraints influencing epidermal cell fate may shed new light on the issue.

## Author contributions

Shinobu Takada wrote the main manuscript text and Hiroyuki Iida prepared Figure 1 and Supplementary Table 1. All authors reviewed the manuscript.

### Conflict of interest statement

The authors declare that the research was conducted in the absence of any commercial or financial relationships that could be construed as a potential conflict of interest.
